# Real world study in Italian public hospital with Efgartigimod in patients affected by generalized myasthenia gravis: influence of clinical and serological factors

**DOI:** 10.3389/fneur.2025.1555068

**Published:** 2025-05-07

**Authors:** Manlio Sgarzi, Paolo Paone, Giorgia Camera, Emanuela Agazzi, Sara Mazzoleni, Francesca Martorana, Dario Alimonti

**Affiliations:** ASST Papa Giovanni XXIII Hospital, Bergamo, Italy

**Keywords:** Myasthenia Gravis, Efgartigimod, Thymectomy, T cells, AChR

## Abstract

**Background:**

Myasthenia gravis (MG) is an autoimmune neuromuscular disorder caused by IgG autoantibodies targeting the neuromuscular junction. Recycling of IgG is mediated by the neonatal Fc receptor (FcRn). Efgartigimod, an Fc fragment of human IgG1, has demonstrated efficacy in MG; however, the clinical characteristics of patients with the highest response remain unclear.

**Methods:**

Twelve patients with AChR-positive generalized MG were treated with two cycles of Efgartigimod over 1 year, and nine patients completed a third cycle. Clinical evaluation was conducted using MG-ADL at four time points and QMG at the beginning and end of each cycle. MG-ADL and QMG scores were further subdivided into ocular (O), bulbar (B), and generalized (G) symptom subdomains, and patients were classified as predominantly ocular (pO), bulbar (pB), or generalized (pG) based on symptom prevalence.

**Results:**

Significant improvements were observed in MG-ADL and QMG from baseline across all symptom subdomains. Baseline AChR antibody levels correlated with MG-ADL improvement (*p* < 0.04). Thymectomized patients demonstrated superior outcomes, with MG-ADL improving by 62% versus 22% (*p* < 0.01) and QMG by 45% versus 3.5% (*p* < 0.01) during the first two cycles. Patients with pO symptoms responded less to therapy, with generalized symptoms contributing most to the minor response.

**Discussion:**

Our findings suggest that patients with high baseline AChR antibody titers, previous thymectomy, and non-ocular symptom predominance respond better to Efgartigimod. These results underscore the need for larger studies to validate these observations and optimize patient selection.

## Introduction

**Figure 1:**
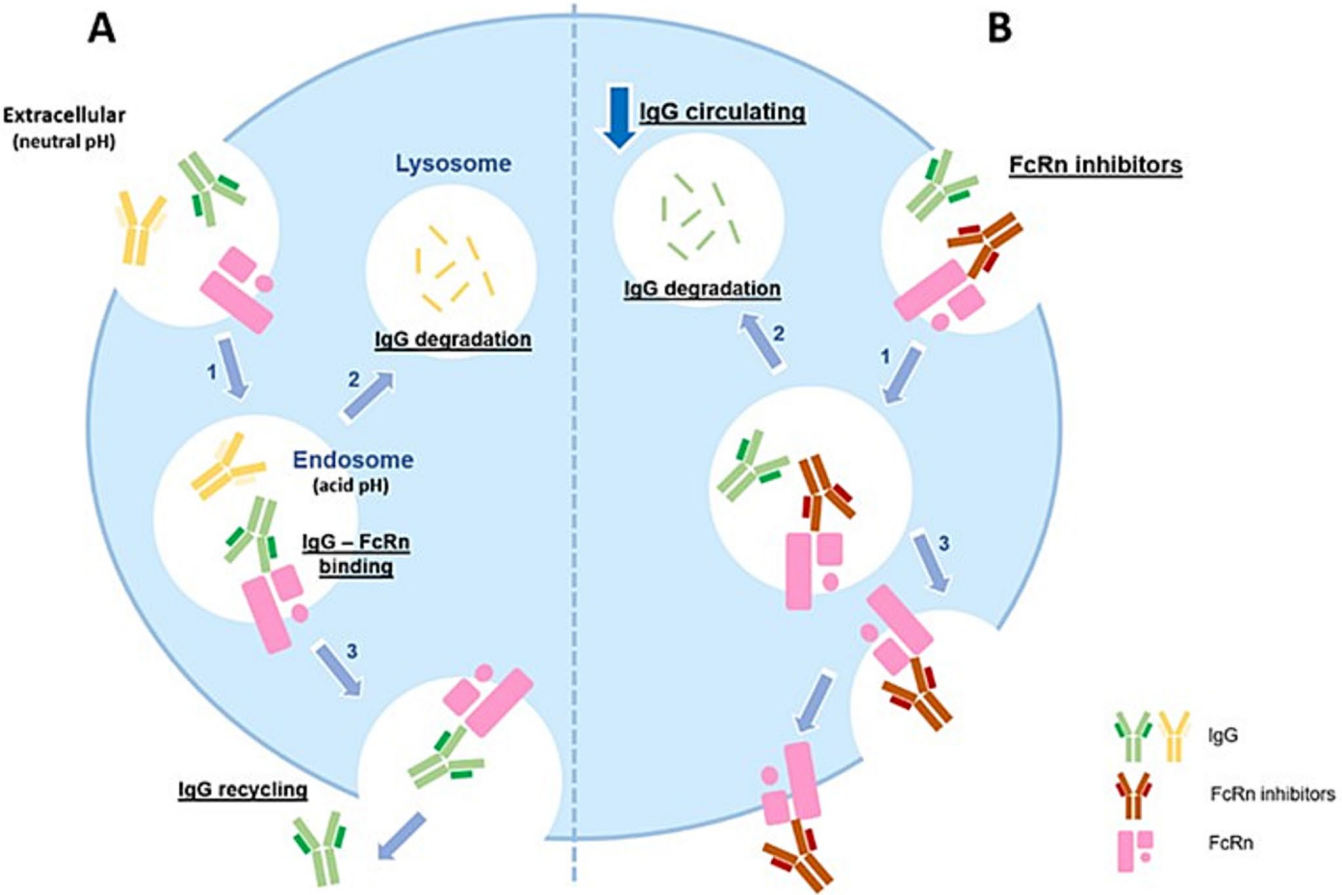
FcRn, Neonatal Fc receptor; IgG, immunoglobulin G. **(A)** FcRn is widely expressed on endothelial cells. IgG enters the cell via fluid-phase endocytosis. IgG bind to FcRn in acidic conditions (pH 6.0–6.5) to form an IgG-FcRn complex (1). IgG that do not bind to FcRn in the acidified body are degraded in the lysosome (2). Under physiological conditions (extracellular compartment, pH 7.4), the IgG-FcRn complex dissociates at the cell surface and the IgG gets released again into the blood circulation (3). By this mechanism, FcRn efficiently safeguards IgG from lysosomal degradation, thus increasing the half-life of IgG. **(B)** FcRn inhibitors are monoclonal antibodies with higher affinity and an increased binding capacity to FcRn at neutral and acidic pH (1). Inside the cell, FcRn inhibitors compete with IgG for binding to FcRn, preventing IgG binding to FcRn. The unbound IgG enter the lysosomes where they are degraded (2). The process results in reduced IgG levels in the circulation, including the pathogenic IgG antibodies.

Myasthenia gravis (MG) is an autoimmune disorder characterized by impaired neuromuscular transmission. As a prototypical B cell-mediated autoimmune disease, MG is caused by IgG autoantibodies with well-established pathogenic effects (1). The half-life of circulating human IgG ranges between 3 and 4 weeks. This prolonged circulation is not solely dependent on synthesis but is largely attributed to continuous salvage and recycling mechanisms mediated by the neonatal Fc receptor (FcRn) (2). FcRn, a molecule structurally similar to MHC class I, plays a pivotal role in maintaining IgG and albumin levels by preventing their lysosomal degradation. This recycling mechanism extends the half-life of IgG approximately fourfold compared to other immunoglobulins not recycled by FcRn, such as IgM or IgA. During cellular uptake, the Fc region of IgG binds to two FcRn molecules under acidic endosomal conditions. This interaction prevents lysosomal degradation, allowing IgGs to be recycled and released at physiological pH into the extracellular environment. Consequently, FcRn perpetuates the availability of autoantibodies in IgG-mediated diseases, including generalized myasthenia gravis (gMG). The presence of pathogenic autoantibodies is a hallmark of autoimmune disorders, especially for myasthenia gravis (MG). Current therapies, such as plasmapheresis, IVIG infusions, and other immunosuppressive agents, aim to reduce pathogenic antibodies. However, most of these therapies have broader mechanisms of action and significant side effects. In virtue of a better understanding of the molecular structure and its biological properties, FcRn has emerged as an interesting target in the treatment of myasthenia gravis and other autoimmune disorders, as it allows the reduction of autoantibodies by blocking FcRn. By blocking FcRn, the catabolism of all subclass of IgG is inceased (Figure 1). This mechanism could be useful in various autoimmune disorders, as the degradation of IgG can be induced by blocking FcRn receptors, making it a rational therapeutic approach. Several drugs exploit this mechanism of action such as Rozanolixizumab, a human IgG4 anti-FcRn antibody, while Nipocalimab or Batoclimab, are fully humanized monoclonal antibodies of the IgG1 class (3).

Efgartigimod (EFG), an Fc fragment of the human IgG1 antibody, has been engineered to exhibit greater affinity for FcRn than endogenous IgG, while retaining pH-dependent binding characteristics. By competitively binding to FcRn, Efgartigimod facilitates the degradation of unbound endogenous IgG in the acidic intracellular environment (4). Efgartigimod has demonstrated efficacy across various MG subgroups and severity levels (5), leading to its approval for managing gMG. Despite these advancements, the optimal dosage, duration of treatment, and identification of patient subpopulations most likely to benefit require further investigation. Here, we report our experience with the clinical efficacy and outcomes of Efgartigimod treatment in patients managed at a public hospital.

## Method

The Expanded Access Program (EAP - version 1.0) was proposed with the logic of providing access to EFG to gMG patients before regulatory approval (GENERATIVE protocol version 1.0, September 10, 2021). All patients, after being adequately informed about the expected benefits and risks of the treatment, signed the informed consent before enrollment in the GENERATIVE program. The enrollment of each patient was possible after the approval of the EAP by the ethics committee of the ASST Papa Giovanni XXIII of Bergamo and subsequently the treatment with Efgartigimod was authorized by the Italian Medicines Agency with resolution in GU n.160 of 11-7-2023.

### Inclusion criteria

In GENERATIVE protocol procedures, the following inclusion criteria had to be met:

- > 18 years at the time of signing the informed consent.
- Patients diagnosed with MG (AChR-Ab seropositive or seronegative) with generalized muscle weakness supported by one of the following factors:
  - Presence of AChR-Ab.
  - Abnormal neuromuscular transmission (single fiber or repeated stimulation).
  - History of positive Edrophonium Chloride test.
  - Demonstrated improvement in MG symptoms, after using oral anticholinesterase inhibitors.
- Myasthenia Gravis Foundation of America (MGFA) Classification II, III, IVa or IVb (6).
- Total Myasthenia Gravis Activity of Daily Living (MG-ADL) score of ≥ 5 points, with > 50% of the total score attributed to non-ocular symptoms, or at least two non-ocular items had to have a score of ≥ 2.
- Patient has been vaccinated against COVID-19 or had a negative COVID-19 test result within 2 weeks prior to enrollment.
- Total IgG ≥ 6 g/L within 1 month of screening.

### Treatment

EFG was administered intravenously at a dose of 10 mg/kg as a 1-h infusion in cycles of four weekly infusions. The initial fixed period included two 3-weeks cycles (four infusions per cycle T0-T1-T2-T3), each followed by a 4-weeks fixed wash-out period. Subsequently, in case of worsening of MG-ADL (7) and Quantitative Myasthenia Gravis (QMG) (8), patients could enter the flexible period at the discretion of the doctor, who could prescribe a new cycle of four infusions, always after a wash-out of at least 4 weeks, monitoring during the wash-out phase whether the clinical conditions were suitable, to prescribe further treatment. Throughout the period observed in this study, the pharmacological treatments of patients with non-steroidal immunosuppressants (NSIT), cholinesterase inhibitors and corticosteroids did not change.

### Clinical evaluation of MG

Clinical evaluation was performed according to the following clinical scales specific for MG:

- MG-ADL
- QMG

MG-ADL scores were administered before each infusion in the first 3 administrations of each cycle and after the end of the last infusion of each cycle. The QMG score was recorded before the first and after the fourth infusion of each cycle. MG-ADL improvement was defined as a reduction of at least 2 points in the score; QMG improvement was defined as a reduction of at least 3 points in total QMG scores (1–4, 9, 10). An increase of at least 2 points for the MG-ADL score and 3 points for the QMG scores was considered clinical criterion for prescribing EFG retreatment during clinical follow-up in the flexible period. The improvements in MG-ADL and QMG were calculated by comparing the score of the last time point of each cycle (T3) with that recorded before the first infusion (T0), as a percentage. Furthermore, to obtain more specific data on the improvement obtained by patients before and after the different cycles of EFG infusion, we divided the total raw scores of MG-ADL and QMG into subclasses for specific symptoms (11), Ocular (O), Bulbar (B) and Generalized (G), adding the scores of the relevant Items (Table 1).

**Table 1:** MG-ADL and QMG subdomains items.

Assessment	Ocular items	Possible score	Bulbar items	Possible score	Generalized items	Possible score
MG-ADL	Diplopia, Ptosis	0–6	Speech Swallowing Chewing	0–9	Ability to brush Ability to arise Breathing	0–9
QMG	Diplopia Ptosis Facial muscles	0–9	Speech Swallowing	0–6	Hand Grip L/R Limb strength arms/legs Head lift Forced Vital Capacity	0–24

### Statistical analysis

Statistical analyses refer to the first three cycles of therapy with Efgartigimod, given the small number of the fourth cycle. Continuous variables were represented by Mean and Standard Deviation while categorical variables were represented by frequencies and percentages. The clinical efficacy of treatment with EFG for all patients in the study was verified by analyzing the mean of MG-ADL and QMG scores between the times of each cycle, we also verified the same means between T0 of the first cycle (baseline) and T3 of the second (1st-2nd Cycle) and third (1st-3rd Cycle) cycle, with repeated measures ANOVA test and *Post Hoc* analysis with the Bonferroni method. For clinical efficacy at the same times between subgroups of patients, we used the Kruskal-Wallis non-parametric test and U-Mann–Whitney test. ANCOVA was used to verify whether there are relationships between demographic and clinical variables at baseline and percentage improvements in clinical measurement scales.

## Results

### Participants

Twelve patients with AChR antibody-positive generalized myasthenia gravis (gMG) were included in this protocol. In addition to collecting demographic and pharmacological data at baseline, we categorized patients based on symptom predominance at T0 using the MG-ADL scale, as outlined in Table 1. For each patient, the scores for the three symptom types—Ocular (O), Bulbar (B), and Generalized (G)—were divided by their respective maximum possible scores, yielding a percentage impairment for each category. The highest percentage determined the predominant symptom type, classifying patients as predominantly Ocular (pO), predominantly Bulbar (pB), or predominantly Generalized (pG). All 12 patients tested positive for AChR antibodies and were classified as MGFA class II. At baseline (Table 2), three patients were male, with an average age of 58.6 years and an average disease duration of 10.2 years. Their total MG-ADL and QMG scores averaged 7.5 and 12.8 points, respectively. Seven patients had undergone thymectomy, including three with thymoma. Regarding pharmacological therapy, 10 patients were receiving steroids at an average dose of 18.8 mg/day, five were on non-steroidal immunosuppressants (NSIT), and eight were taking acetylcholinesterase inhibitors (AChEI). Four patients were on both steroids and NSIT, and one patient was on AChEI therapy alone. In the 3 months prior to the first Efgartigimod infusion, seven patients required treatment with immunoglobulins or plasmapheresis (IVIg or PLEX). Based on the highest symptom subclass score, four patients each were classified as pO, pB, and pG.

**Table 2:** Patient characteristics.

Characteristics of the patients at baseline	mean / *n*	sd / %
Demographics		
Male (*n*)	3	25,0%
Age (y)	58.6	14.0
Disease duration (y)	10.2	6.5
MG-ADL total score	7.5	2.6
QMG Total score	12.8	4.1
AChR Antibody levels (nmol/L)	52.7	55.1
Thymectomy (*n*)	7	58.3%
Thymoma (*n*)	3	25.0%
MG Therapy		
Any Steroid (*n*)	10	83.3%
Any Steroid (mg)	18.8	10.5
Any NSIT (*n*)	5	41.7%
Any AChEI (*n*)	8	66.7%
IVIg /Plex (*n*)	7	58.3%
Steroid+NSIT (*n*)	4	33.3%
AChEI only (*n*)	1	8.3%
Type of patients		
pO (*n*)	4	33.3%
pB (*n*)	4	33.3%
pG (*n*)	4	33,3%

*n, frequency; sd, standard deviation; y, yars; NSIT, Non-steroidal immunosuppressants treatments; AChEI, acetylcholinesterase inhibitor; IVIg, immunoglobulins in vein; Plex, plasmapheresis; pO, predominantly Ocular; pB, predominantly Bulbars; pG, predominantly Generalized.*

### Clinical assessments in all participants

**Figure 2:**
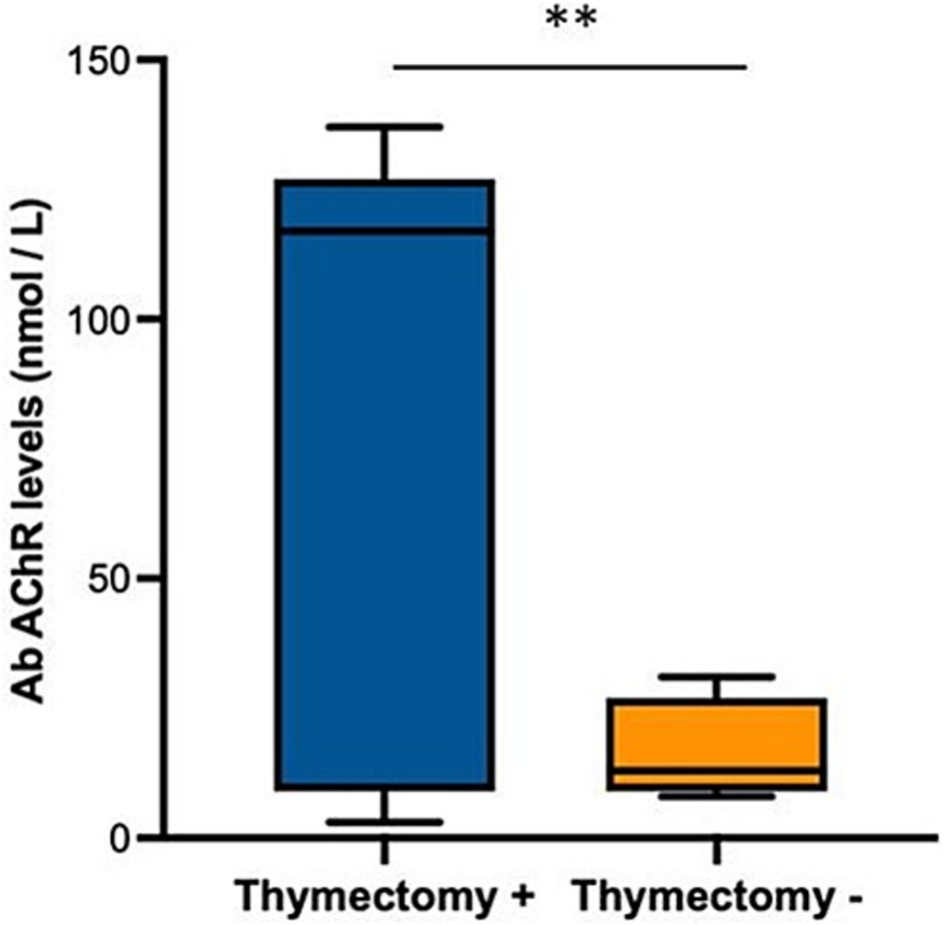
Box plot showing anti-AChR antibody levels, expressed as nmol / L, in patients who underwent (positive, +) or not (negative, −) thymectomy. ** *p* < 0.01, U-Mann Whitney for indipendent samples.

Twelve patients who started treatment with Efgartigimod completed both the first and second cycles of infusions with a 4-weeks interval between the two cycles as per protocol. Nine patients started the third cycle of therapy with Efgartigimod at an average of 55.1 ± 40.0 days after the end of the second cycle and completed it, while only 3 of these patients started the fourth cycle at an average of 35.3 ± 5.4 days from the end of the third cycle. Considering all study participants, the means of MG-ADL and QMG were statistically significantly reduced between T0 and T3 of each infusion cycle, considering that in the third cycle for both clinical measurements there were nine patients. The improvements in the MG-ADL and QMG scores were statistically significant both within and between all cycles, as reported in Table 3. In our cohort, baseline AChR antibody levels were markedly higher in patients who had undergone thymectomy compared to those who had not (78.19 ± 45 vs. 16.94 ± 14 nmol/L, respectively; *p* < 0.01, Figure 2).

**Table 3:** Mean and standard deviation of MG-ADL and QMG scores at the beginning and at the end of each cycle of therapy with Efgartigimod.

MG-ADL assessment in all patients											
	1° Cycle		2° Cycle		3° Cycle		*p* value*				
MG-ADL	T0	T3	T0	T3	T0	T3	1° Cycle	2° Cycle	3° Cycle	1°-2° Cycle	1°-3° Cycle
mean	7.5	4.0	5.8	3.1	7.7	4.0	0.007	0.010	0.0009	0.0003	0.016
SD	2.6	2.7	4.1	1.5	3.4	2.8					

QMG assessment in all patients											
	1° cycle		2° cycle		3° cycle		*p* value*				
QMG	T0	T3	T0	T3	T0	T3	1° Cycle	2° Cycle	3° Cycle	1°-2° Cycle	1°-3° Cycle
mean	12.8	9.2	12.2	9.3	13.0	10.1	0.010	0.035	0.004	0.007	0.043
SD	4.1	4.7	6.8	4.3	4.4	4.8					

* Repeated measures ANOVA with *Post Hoc* analysis using Bonferroni method.

**Figure 3:**
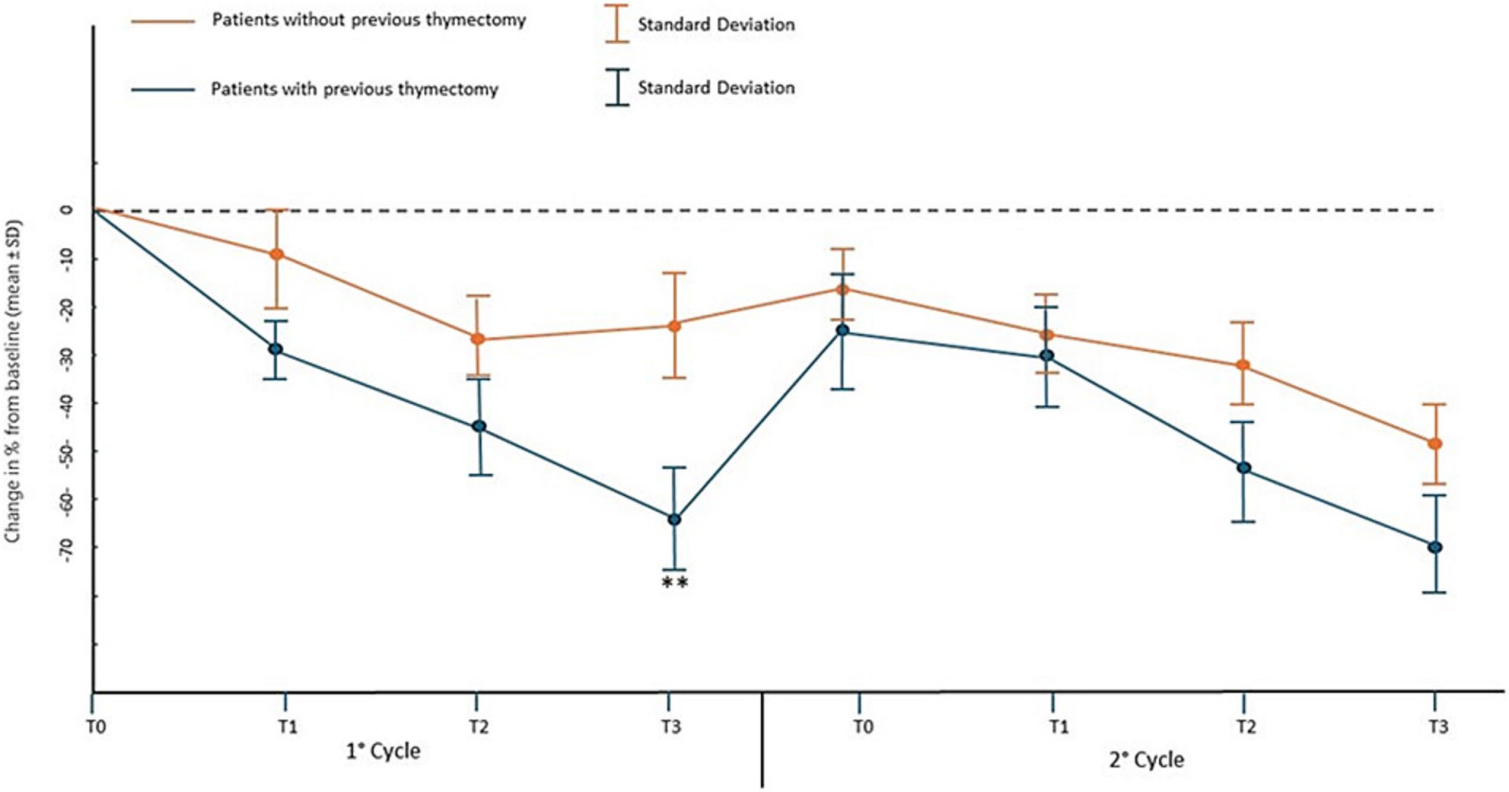
Percentage change in mean MG-ADL score and standard deviation from baseline (T0 of the 1st cycle) at the time points of the first two EFG cycles in patients who have or have not previously undergone thymectomy.** *p* < 0.01 with U-Mann–Whitney test.

**Figure 4:**
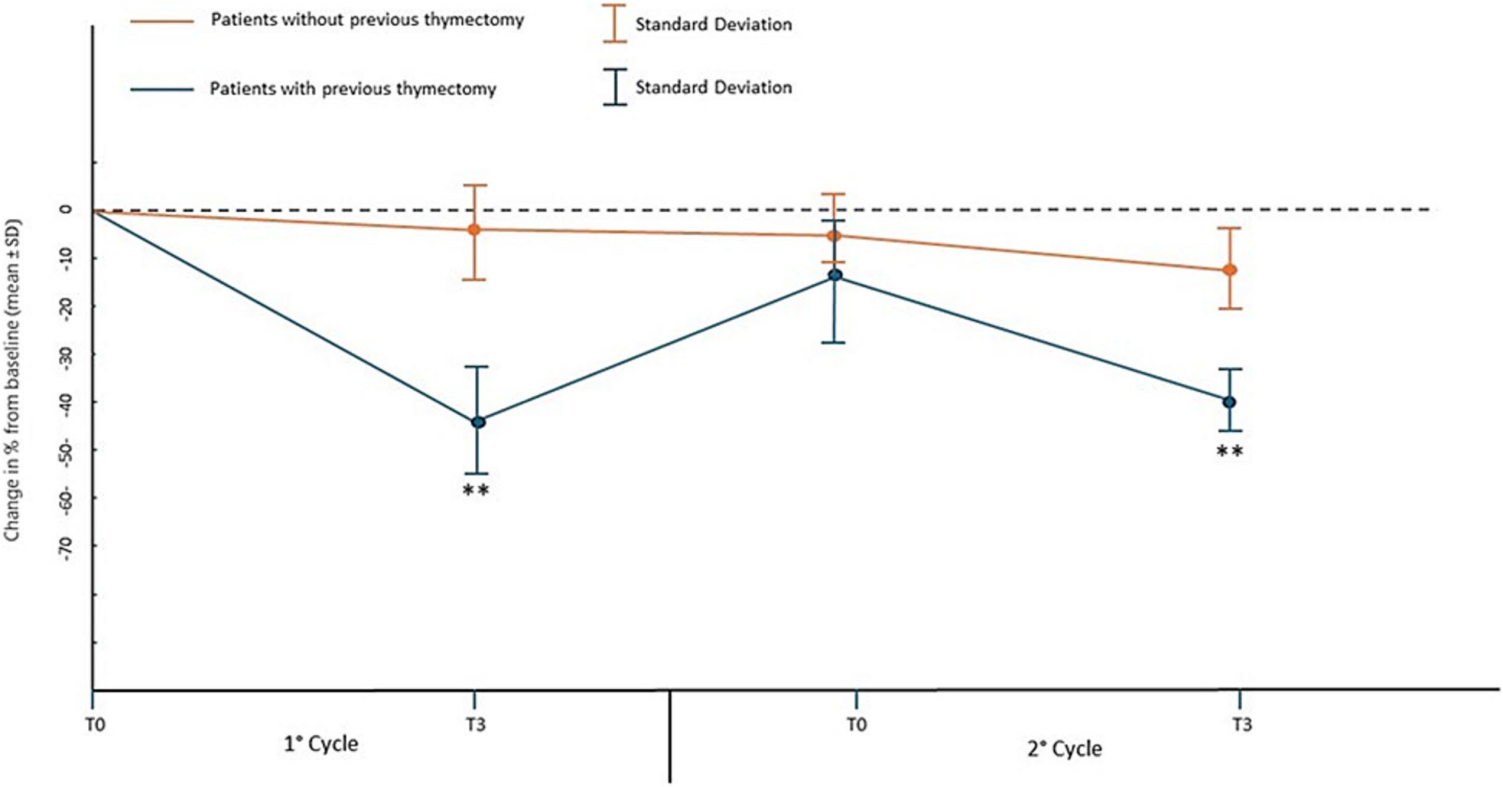
Percentage change in mean QMG score and standard deviation from baseline (T0 of the 1st cycle) at the time points of the first two EFG cycles in patients who have or have not previously undergone thymectomy.** *p* < 0.01 with U-Mann–Whitney test.

Covariate analysis showed a significant direct proportional relationship between the level of AChR antibodies at baseline and the improvement in MG-ADL (*p* < 0.04) and QMG (*p* < 0.01) between the beginning and the end of the first cycle of infusions, while in the second and third cycle this relationship disappeared. Furthermore, patients who had undergone thymectomy despite having had the surgical procedure several years before baseline (median 6.3 years range 4.3–8.8), the improvements between the beginning and the end of the first cycle of infusions in MG-ADL were significantly higher with the mean score ranging from 8.0 ± 2.8 to 3.1 ± 3.0 (which in percentage terms is a change of 62% from baseline *p* < 0.01, Figure 3), compared to the other patients who had a mean score ranging from 6.8 ± 3.1 to 5.2 ± 2.6 (which in percentage terms is a change of 22% from baseline). After the end of the second cycle, the significant difference between the improvements in MG-ADL score between patients with previous thymectomy and the others, disappeared. Improvements in QMG between the start and end of the first infusion cycle in patients with prior thymectomy were significantly greater with a mean score ranging from 13.4 ± 4,7 to 7.4 ± 4.2 (change of 45% from baseline *p* < 0.01, Figure 4), compared to other patients who had a mean score ranging from 12.0 ± 5.0 to 11.6 ± 4.5 (change of 3.5% from baseline). After the end of the second cycle, this difference remained significantly different, with QMG scores decreasing from 13.4 ± 4.7 at baseline to 8.1 ± 4.1 at T3 of the second cycle (change of 40% from baseline *p* < 0.01) for patients with prior thymectomy and from 12.0 ± 5.0 at baseline to 10.9 ± 4.7 (change of 9% from baseline) at T3 of the second cycle, for patients who had not undergone a previous thymectomy.

### Ocular signs in all patients

The analysis within and between treatment cycles in ocular signs showed a significant reduction in the average MG-ADL score only between the beginning and the end of the second cycle of infusions. We observed that from baseline to the end of the second and third cycle, the decrease in the MG-ADL score was statistically significant. In QMG, the improvement in ocular signs was significant between baseline and the end of the first and third cycles (*p* = 0.05, *p* = 0.01, Table 4).

**Table 4:** Mean and standard deviation of MG-ADL and QMG scores in Ocular signs at the beginning and at the end of each cycle of therapy with Efgartigimod.

Ocular signs in MG-ADL assessment in all patients											
	1° Cycle		2° Cycle		3° Cycle		*p* value*				
MG-ADL	T0	T3	T0	T3	T0	T3	1° Cycle	2° Cycle	3° Cycle	1°-2° Cycle	1°-3° Cycle
mean	2.22	1.56	2.00	1.11	1.80	1.40	0.28	0.044	0.34	0.036	0.031
SD	1.39	1.51	1.73	1.16	1.44	1.56					

Ocular signs in QMG assessment in all patients											
	1° Cycle		2° Cycle		3° Cycle		*p* value*				
QMG	T0	T3	T0	T3	T0	T3	1° Cycle	2° Cycle	3° Cycle	1°-2° Cycle	1°-3° Cycle
mean	2.88	1.55	2.44	2.00	2.40	1.00	0.050	0.16	0.10	0.19	0.01
SD	1.87	1.23	2.00	1.78	1.23	1.32					

* Repeated measures ANOVA with *Post Hoc* analysis using Bonferroni method.

### Bulbar signs in all patients

The analysis within and between treatment cycles in the bulbar signs showed a significant decrease in the average of the MG-ADL score between the beginning and the end of the first and second cycles and also between baseline and the end of the second and third cycles. The mean QMG score of the bulbar signs decreased significantly only between the beginning and the end of the first cycle and also between baseline and the end of the second and third cycle (*p* = 0.041, *p* = 0.034, Table 5).

**Table 5:** Mean and standard deviation of MG-ADL and QMG scores in Bulbar signs at the beginning and at the end of each cycle of therapy with Efgartigimod.

Bulbar signs in MG-ADL assessment in all patients											
	1° Cycle		2° Cycle		3° Cycle		*p* value				
MG-ADL	T0	T3	T0	T3	T0	T3	1° Cycle	2° Cycle	3° Cycle	1°-2° Cycle	1°-3° Cycle
mean	3.88	1.13	2.13	0.88	3.20	1.20	0.030	0.010	0.11	0.012	0.021
SD	2.00	1.66	1.23	1.88	1.58	1.54					

Bulbar signs in QMG assessment in all patients											
	1° cycle		2° cycle		3° cycle						
QMG	T0	T3	T0	T3	T0	T3	1° Cycle	2° Cycle	3° Cycle	1°-2° Cycle	1°-3° Cycle
mean	2.50	0.50	1.43	0.86	2.40	0.60	0.018	0.10	0.12	0.041	0.034
SD	1.79	1.39	1.49	1.43	2.00	0.83					

* Repeated measures ANOVA with *Post Hoc* analysis using Bonferroni method.

### Generalizated signs in all patients

The analysis within and between treatment cycles in generalized signs showed a significant decrease in the mean MG-ADL score only in the first infusion cycle. The average QMG score of generalized signs showed a significant decrease between T0 and T3 in the second cycle and between baseline and T3 of second cycle (*p* = 0.016, Table 6).

**Table 6:** Mean and standard deviation of MG-ADL and QMG scores in Generalized signs at the beginning and at the end of each cycle of therapy with Efgartigimod.

Generalized signs in MG-ADL assessment											
	1° Cycle		2° Cycle		3° Cycle						
MG-ADL	T0	T3	T0	T3	T0	T3	1° Cycle	2° Cycle	3° Cycle	1°-2° Cycle	1°-3° Cycle
mean	3.20	1.90	3.40	2.20	5.25	3.00	0.03	0.07	0.11	0.10	0.45
SD	1.44	1.73	2.11	1.89	2.30	2.07					

Generalized signs in QMG assessment											
	1° Cycle		2° Cycle		3° Cycle						
QMG	T0	T3	T0	T3	T0	T3	1° Cycle	2° Cycle	3° Cycle	1°-2° Cycle	1°-3° Cycle
mean	9.22	7.89	9.11	7.22	9.66	9.50	0.065	0.016	0.89	0.020	0.87
SD	2.54	3.55	5.28	3.99	3.80	4.70					

* Repeated measures ANOVA with *Post Hoc* analysis using Bonferron method.

### MG-ADL and QMG subdomains for type of patients

We observed that the percentage of improvement of MG-ADL from baseline to T3 of second cycle, was not different between the patient types of patients, while there was a significant difference in the percentages of improvement of QMG. In the pO patient group, the percentage of improvement of the individual subdomains of QMG, although with a small sample size for each subdomain, showed differeces, with Generalized Signs being the least responsive symptomatology to treatment (Table 7).

**Table 7:** Percentage (%) of improvement in MG-ADL and QMG by patient type and symptom subdomain (sd) * Kruskal-Wallis non-parametric test.

	Prevalent ocular patients			*p*	Prevalent bulbar patients			*p*	Prevalent generalizated patients			*p*
% improvement MG-ADL		59.6 (31)				50.11 (40)				61.02 (48)		0.89*
	Ocular signs	Bulbar signs	Generalized signs		Ocular signs	Bulbar signs	Generalized signs		Ocular signs	Bulbar signs	Generalized signs	
	55 (33)	39 (31)	66 (28)	0.74**	44 (48)	49 (33)	57 (31)	0.67**	39 (53)	66 (41)	65 (44)	0.39**

	Prevalent ocular patients			*p*	Prevalent bulbar patients			*p*	Prevalent generalizated patients			*p*
% improvement QMG		13.42 (19)				37.44 (20)				28.32 (51)		0.03*
	Ocular signs	Bulbar signs	Generalized signs		Ocular signs	Bulbar signs	Generalized signs		Ocular signs	Bulbar signs	Generalized signs	
	44 (22)	45 (27)	5 (13)	0.01**	32 (15)	46 (32)	39 (22)	0.45**	59 (41)	78 (76)	66 (37)	0.34**

** U-Mann–Whitney test for independent samples.

## Discussion

During the Generative protocol, we treated 12 patients with AChR-positive generalized myasthenia gravis (gMG), all of whom received two complete cycles of Efgartigimod infusions between August 2023 and August 2024. Among these, nine patients completed a third cycle, and four continued with therapy, undergoing up to six cycles almost chronically, in line with the EAP protocol. Due to the limited number of patients beyond the third cycle, we focused our analysis on data from baseline to the end of the third cycle. Our study aimed to identify the clinical characteristics of patients who experienced the best outcomes with Efgartigimod, to guide future treatment decisions. Across all 12 patients, we observed strong evidence of the therapy’s effectiveness, with significant improvements in MG-ADL and QMG scores from baseline to the end of every cycles. Efgartigimod, an FcRn antagonist, induces a profound and sustained reduction in serum IgG levels, significantly alleviating symptoms of gMG (12). In our cohort, no adverse effects were observed. Clinical improvements correlated positively with baseline AChR antibody levels, particularly during the first cycle, for both MG-ADL and QMG assessments. The use of therapeutic molecules targeting the neonatal Fc receptor (FcRn) aims at the selective removal of immunoglobulin G (IgG), a process that is also widely performed by plasmapheresis in patients with myasthenia gravis (MG). It has been observed that high levels of anti-AChR antibodies at baseline are one of the favorable prognostic factors for a better response to treatment (12). In a 2022 review, the authors questioned the correlation between autoantibody levels and disease severity in individuals with myasthenia gravis (MG) (13) and in most of the studies reviewed, positive and significant correlations between autoantibody levels and clinical improvement were described (13). Another recent review (14) corroborates these findings, noting that the highest antibody titers occur in patients with thymic hyperplasia, followed by intermediate levels in those with thymoma, and lower levels in individuals with atrophic or normal thymus. Although antibody levels were correlated with clinical improvements in the first cycle, this relationship decreased in subsequent cycles. This trend may reflect a possible reduction in antibody levels after initial treatments with Efgartigimod (15) and similar therapies (16). Patients who had undergone thymectomy, even if the surgical procedure had been performed at least 4.3 years before and the positive effect of thymic surgery occurs within a maximum of 1–2 years from the event (17), seemed to benefit more from Efgartigimod, with improvements in MG-ADL scores after the first cycle and QMG improvements sustained also during the second cycle appeared to benefit more from Efgartigimod, with greater improvements in MG-ADL scores after the first cycle and sustained QMG improvements through the second cycle. This may be linked to the inflamed thymus in myasthenia gravis, particularly in cases of thymoma, which is often infiltrated with B cells and Th cells, including Th-GM and Th-CD103 subtypes (18). These T cells, known to drive chronic pathogenic antibody production, can persist even after thymectomy. Long-term studies suggest that thymectomy does not fully resolve symptoms, particularly in younger patients, who may continue to struggle with muscle weakness and fatigue (19, 20). In these cases, Efgartigimod’s mechanism—blocking FcRn and reducing pathogenic antibodies—might be especially valuable. Our findings also showed significant improvements across all MG-ADL subdomains (ocular, bulbar, generalized) and in QMG composite scores. However, prior studies have reported varying degrees of improvement in specific subdomains depending on the treatment (11, 21). In our cohort, patients with predominantly ocular symptoms (pO) showed less improvement (13.42% in QMG scores) compared to those with bulbar (pB, 38.44%) or generalized symptoms (pG, 71.89%). This suggests that pO patients may respond less robustly to Efgartigimod, potentially due to distinct underlying mechanisms or reduced involvement of pathogenic antibodies in ocular symptoms (22, 23). Although our sample is small, our real-world observations suggest that patients with elevated AChR antibody titers and a history of thymectomy may respond more favorably to Efgartigimod. The relationship between the predominance of non-ocular symptoms and a better clinical outcome, while observed, does not allow for any significant conclusions given the small number of patients in the pO, pB, and pG subgroups. The main limitation of this study is undoubtedly the small number of participants, which makes the results less robust, although it provides significant observations that may encourage future research, especially real world, on potentially more treatment-responsive target patients, the long-term effects of Efgartigimod, and the optimization of dosing regimens. Furthermore, it should be noted that participants presented moderate weakness at the beginning of the study (MGFA class II), which may have influenced the clinical observations, especially in evaluating the responses of the patient subgroups given the small sample sizes. Finally, given the results of previous clinical trials and in the absence of a control group, assessments of the response to Efgartigimod therapy must be considered with due caution, especially in the early stages of treatment (24, 25).

## Data Availability

The raw data supporting the conclusions of this article will be made available by the authors, without undue reservation.
